# Impact of Different Packaging Configurations on A Topical Cream Product

**DOI:** 10.1007/s11095-024-03772-5

**Published:** 2024-09-30

**Authors:** Yousuf H. Mohammed, S. N. Namjoshi, K. C. Telaprolu, N. Jung, H. M. Shewan, J. R. Stokes, H. A. E. Benson, J. E. Grice, S. G. Raney, E. Rantou, Maike Windbergs, Michael S. Roberts

**Affiliations:** 1 Therapeutics Research Centre, Frazer Institute, Faculty of Medicine, The University of Queensland, Brisbane, Australia; 2 School of Pharmacy, The University of Queensland, Woolloongabba, QLD 4102 Australia; 3 Institute of Pharmaceutical Technology, Goethe University, Frankfurt, Germany; 4 School of Chemical Engineering, The University of Queensland, Brisbane, QLD 4072 Australia; 5 Curtin Medical School, Curtin University, Perth, WA Australia; 6 School of Pharmacy and Medical Sciences, University of South Australia, Adelaide, Australia; 7 Therapeutics Research Centre, Basil Hetzel Institute for Translational Medical Research, The Queen Elizabeth Hospital, Adelaide, Australia; 8 Office of Research and Standards, Office of Generic Drugs, Center for Drug Evaluation and Research, United States Food and Drug Administration, Silver Spring, MD USA; 9 Office of Biostatistics, Office of Translational Sciences, Center for Drug Evaluation and Research, United States Food and Drug Administration, Silver Spring, MD USA; 10 School of Pharmacy, The University of Queensland, Brisbane, 2102 Australia

**Keywords:** Bioequivalence, Compliance, Confocal Raman microscopy, Cryo-SEM, IVPT, Microstructure, Pump dispensers, Physicochemical and structural, Quality attributes of semisolids, Q3, Rheology

## Abstract

**Purpose:**

The objective of this study was to investigate whether different dispensing processes can alter the physicochemical and structural (Q3) attributes of a topical cream product, and potentially alter its performance.

**Methods:**

Acyclovir cream, 5% (Zovirax®) is sold in the UK and other countries in a tube and a pump packaging configurations. The structural attributes of the cream dispensed from each packaging configuration were analyzed by optical microscopy, confocal Raman microscopy and cryo-scanning electron microscopy. Rheological behavior of the products was also evaluated. Product performance (rate and extent of skin delivery) was assessed by *in vitro* permeation tests (IVPT) using heat-separated human epidermis mounted in static vertical (Franz-type) diffusion cells.

**Results:**

Differences in Q3 attributes and IVPT profiles were observed with creams dispensed from the two packaging configurations, even though the product inside each packaging appeared to be the same in Q3 attributes. Visible globules were recognized in the sample dispensed from the pump, identified as dimethicone globules by confocal Raman microscopy. Differences in rheological behaviour could be attributed to these globules as products not dispensed through the pump, demonstrated a similar rheological behaviour. Further, IVPT confirmed a reduced rate and extent to delivery across human epidermis from the product dispensed through a pump.

**Conclusions:**

Different methods of dispensing topical semisolid products can result in metamorphosis and Q3 changes that may have the potential to alter the bioavailability of an active ingredient. These findings have potential implications for product developers and regulators, related to the manufacturing and comparative testing of reference standard and prospective generic products dispensed from different packaging configurations.

## Introduction

The design and development of topical pharmaceutical formulations is driven by the goal of optimal product performance, in terms of therapeutic efficacy, physical stability and patient compliance [1]. Topical semisolid products have a complex arrangement of matter that imparts particular physicochemical and structural (Q3) attributes to the dosage form, which modulate the performance of the product. Excipients may remain at the site of administration or penetrate into the skin along with the active ingredient, where they may influence its therapeutic efficacy [1, 2]. There is a large body of literature describing how the performance of topical products can be influenced by the specific components of a formulation, individually, and by the type of vehicle involved (e.g. cream, gel, ointment or foam) which is a function of the composition of components, collectively [3–7]. This knowledge base describes how the qualitative (Q1) components (i.e., the excipients) and the quantitative (Q2) composition (i.e., the formula) of a topical product may influence its bioavailability. However, even pharmaceutically equivalent topical products which have the same amount of the same drug (Q1) in the same dosage form (Q2) (i.e., the same type of vehicle) may exhibit differences in bioavailability, and may not necessarily be bioequivalent [8]. These differences in product performance may be modulated by the arrangement of matter in the formulation (i.e., the Q3 attributes of the product), and the influence of changes to these Q3 attributes which may arise from product metamorphosis during product dispensing and administration would, therefore, be critical to understand. Recent product specific guidelines, for example Acyclovir guidance [9], recommend that e influence of any differences in the container closure systems between the test and RLD products, which may influence the physicochemical properties of the cream when dispensed, should be considered in the design of the physical and structural characterization studies.

Most topical semisolid cream products consist of oleaginous (oily) and aqueous components, where one phase may be stabilized as fine droplets by the presence of a surfactant, forming a complex arrangement of matter that can influence the Q3 properties of the formulation, the bioavailability of the therapeutic active ingredient(s), as well as the sensorial properties of the product; the texture, firmness and spreadability during application are important factors to consider in topical formulations because these properties impact the patient’s positive or negative perceptions of the product. For example, incorporation of silicones in topical semisolid products applied to the skin and hair, which began in the 1950s, led to enhanced product spreadability and hydration of the skin. Furthermore, most topical semisolid formulations display complex flow behavior, including a breakdown of the microstructure in response to high shear forces (e.g., during product application) [10]. A detailed understanding of the microstructure of a cream base is essential to achieve desirable Q3 characteristics that meet a topical product manufacturer’s Quality Target Product Profile (QTPP) and a patient’s perception of cosmetic acceptability. A QTPP is a prospective list of desired quality attributes (QAs) that should be present in the finished product [11, 12]. The QTPP defines and supports the control of numerous attributes of formulation that may be critical to its quality and performance, mitigating the risk of product failure when the product is manufactured within specified acceptance limits. Hence, when the Q3 attributes of a reference standard product are utilized to define the QTPP for a corresponding generic product, it ensures that numerous potential failure modes for product performance are controlled, thereby supporting pharmaceutical equivalence by design [13].

Traditionally, topical semisolid products have been packaged into simple jars and tubes. However, there is an emerging trend toward more sophisticated container closure systems to dispense a product, such as pump container closure systems, which may offer metered doses with improved ease of use, and may be preferred by patients. For instance, Eichenfield *et al*. [14] reported high compliance rates (75–100% of prescribed doses taken) in 95% of patients using a tretinoin gel microsphere formulation dispensed from a pump over a 12-week period. Although there was no comparison to an alternative packaging configuration (like a tube), patient satisfaction was high, with 82.3% of patients rating the pump container closure systems as an "excellent" or "very good" means of dispensing medication, and 86.0% rating their overall satisfaction with the pump treatment application as "very satisfied" or "extremely satisfied" [14]. Similarly, Rueda *et al*. compared a once-daily application of adapalene and benzyl peroxide gel in acne treatment where the subjects used a pump or tube for 7 days then switched to the alternate device [15]. They reported that 79% (n = 230) preferred the pump, reporting that it was easy to use, clean and convenient, with the authors also concluding that it provided a more consistent dose to the skin.

A manufacturer’s choice of product packaging for a topical semisolid dosage form may be based on convention, patient use patterns, or the opportunity for product differentiation from competitors. An important consideration when selecting or changing a packaging configuration is how the shear forces during the dispensing of the formulation from the container closure system (e.g., a tube or a pump) may influence its Q3 attributes, and the performance of the product [15]. The same product may be marketed in different packaging configurations (dispensing systems), as is the case for acyclovir cream, which is available in the UK packaged in both container closure systems, a simple tube, and a pump. There has been little published literature on the effect of the dispensing system on the Q3 attributes of topical formulations or their performance, and there may be an assumption by patients, physicians, and pharmacists, that the therapeutic performance is equivalent when a product is dispensed from different packaging configurations. However, if the packaging configurations alter the Q3 attributes of the formulation in different ways during dispensing, and consequently, produce differences in the rate and extent of bioavailability, then it is possible that the therapeutic performance could be altered. Evidence to support this proposition comes from extrusion studies, which show that the microstructure of an extruded mass is affected by equipment parameters such as the dimensions of the orifice, processing parameters including force and speed, and inherent properties of the material itself, including viscosity and shear history [16, 17]. The same principles can be directly applied to the extrusion and dispensing of a semisolid formulation such as a cream and, therefore, the product microstructure could potentially be influenced by the container closure system geometry and dispensing parameters such as shear stress.

Based upon these considerations, the main objective of this work was to investigate the influence of the dispensing process from different packaging configurations on the Q3 attributes and topical bioavailability of an active ingredient in a topical semisolid drug product. For this purpose, a commercial acyclovir cream, 5% (Zovirax® cream marketed in the UK), dispensed from either a tube or a pump, was systematically investigated by optical microscopy, confocal Raman microscopy and cryo-scanning electron microscopy. The distribution of individual components and rheological characteristics of the product dispensed from the tube and the pump (both before and after pump dispensing) were determined. The rate and extent of topical bioavailability was assessed by an *in vitro* permeation test (IVPT) using static, vertical (Franz-type) diffusion cells using heat separated human epidermis. Specifically, the rate and extent of acyclovir bioavailability for the products dispensed from the tube *vs*. the pump was compared based upon cutaneous pharmacokinetics endpoints, and the IVPT study results were compared by a Scaled Average Bioequivalence (SABE) statistical analysis.

## Materials and Methods

### Materials

Zovirax® (UK) acyclovir cream, 5% packaged in different container closure systems (pump and tube) were used in the present study. Acetonitrile (batch no: 15050300) was supplied by RCI Labscan Ltd. (Port Adelaide, South Australia) and ammonium acetate was obtained from Anala® MERCK Pty. Ltd, Victoria Australia. Phosphate buffered saline (PBS) was prepared by dissolving 1 pouch of PBS powder, pH 7.4 obtained from Sigma (Lot number: S2BJ4837U) in 1L purified water. The 10 cSt silicone oil was obtained from Cannon Instrument Company® PA, USA.

### Particle Size Analysis and Identification of Globules Using Optical Microscopy

Cream samples were dispensed from the pump or tube onto a glass slide and spread evenly in a thin layer using a glass cover slip. Images were acquired using a Zeiss Primostar optical microscope with a Zeiss AxioCam ERc 5S. Four microscopic images (each displaying approximately 25 particles) were acquired for each sample at 40 × magnification. In each image, particles were manually counted and measured using the AxioVision software (Release 4.8.2) to obtain particle size information that was then imported into Excel to generate the particle size distribution plots. Particle size was determined by the projected area diameter method [18].

A known amount of cream sample was gently mixed with an oil soluble dye, Sudan red, using a glass rod and observed under the microscope. To measure the globule size, samples were prepared as above and four microscopy images (approximately 25 globules per image) were acquired at 100 × magnification. Globules were manually counted and measured using the AxioVision software (Release 4.8.2). This data was exported to Excel to generate the globule size distribution plot. Additional characterizations were performed to corroborate the results.

### Identification of Globule Chemical Composition by Confocal Raman Microscopy

For Raman analysis, samples of the acyclovir formulations from the tube and pump before and after dispensing were evenly spread on a CaF_2_ glass slide and examined using a confocal Raman microscope (alpha300 R^+^, WITec GmbH, Ulm, Germany). A diode laser with a wavelength of 785 nm was adjusted to a power of 20 mW before a 50 × objective (numerical aperture of 0.8). Raman spectra were recorded from 400 to 1780 cm^−1^ with a spectral resolution of 4 cm^−1^. Using a pinhole of 100 µm prevented detection of signals from out-of-focus-regions. Two dimensional scans in the xy-direction were recorded using an acquisition time of 0.2 s per spectrum and a step size of 0.5 µm. Single spectra were recorded using an acquisition time of 10 s and 10 accumulations. The recorded spectra were processed using WITec Project FOUR software (WITec GmbH, Ulm, Germany). Background subtraction using a polynomic fit and cosmic ray removal were performed for all spectra. Scans were further analyzed by hierarchical cluster analysis and subsequent basis analysis to create chemically selective false color images.

### Examination of the Internal Microstructure of Creams with Electron Microscopy

Morphological examination of the internal microstructure of the acyclovir creams in their native form was carried out using a JEOL scanning electron microscope (JSM7100F, JEOL, USA) equipped with a secondary electron detector. For the cryo-SEM, the cream samples were loaded on the cryo-specimen holder and cryo-fixed in slush nitrogen (-210 C), then quickly transferred under vacuum to the cryo-preparation chamber in the frozen state. The frozen cream samples were fractured using an in-built fracture blade and then sputter-coated with platinum for 120 s at 10 mA. The coated samples were moved to the imaging chamber maintained at -145°C, equipped with an anti-contaminator which was maintained at -194°C. The imaging was undertaken at a voltage of 2 kV by collecting the secondary electron signal.

### Rheological Characterization (Influence of Pre-Shear on Rheological Performance of the Cream)

Bulk rheological properties of acyclovir creams were determined using an AR-G2 rheometer (TA Instruments, New Castle, DE, USA) equipped with a smart swap Peltier plate. A 40 mm parallel plate was used and both the upper and lower plates were covered with sandpaper to prevent the occurrence of slip. All tests were conducted at 32°C, simulating human skin temperature, with a gap height of 0.5 mm (wide gap). The procedures carried out were: (i) Shear Sweep Test (Stress Control Mode), of 10 points per decade, within the relevant stress range (10–450 Pa); (ii) Oscillatory Amplitude Sweep, of 3 points per decade at an angular frequency of 6.283 rad/s using strain sweep method from 0.1–100% strain to determine the storage and loss modulus; and (iii) Creep Tests. Triplicate tests were performed on all cream samples. The tube and pump samples were dispensed from the container directly onto the rheometer plates as sample of approximately 0.5 to 1 ml was dispensed by eye. Approximately 0.5 to 1 ml of the ‘no pump sample’ was scooped from the container after the pump and lid were removed. The no pump sample was then transferred gently from the spoon to plate surface. The plates were immediately set to their required gap height and the excess was trimmed from around the plates before the sample surface was covered with 10 cSt silicone oil to prevent loss of volatiles. All samples for all procedures had a pre-shear step included, whereby the creams were pre-sheared at 1 s^−1^ for 60 s to ensure repeatability of measurements.

The thin film (narrow gap) rheological measurements were carried out on a Haake Mars III stress controlled rotational rheometer with a Peltier controlled element (Thermo Fisher Scientific, Waltham, MA). Parallel plate geometry, 35 mm was used with the gap between the plates set at 50 μm. The gap was zeroed at a force of 4 N to account for the squeeze flow of air [19] and results adjusted for gap error using the methods of Davies and Stokes [20] and Kravchuk and Stokes [21]. Tests were performed on each cream before (not pump-dispensed) and after dispensing from their container closure system.

### Evaluation of Tribological Properties

Tribology (friction) was measured on an Anton Paar MCR 503 rheometer with a rheo-tribocell attachment (Anton Paar Australia Pty Ltd, North Ryde, NSW, Australia). A rotating steel ball was placed in contact with the polydimethylsiloxane (PDMS) pins. A load of 2 N was placed on the pins, equivalent to 4.33 N force in the vertical direction and the friction force (F_f_) measured as a function of the entrainment speed (U). The measurement involves sliding only friction. Soft (compliant) surfaces were used to provide low pressure in the contact, which is consistent with *“in-use”* skin contact.

### Quantification of Acyclovir

A Shimadzu high-performance liquid chromatography (HPLC) system equipped with a binary solvent pump, autosampler, photodiode array detector, thermostatted column compartment and LC solutions chromatographic software was used to measure the concentration of acyclovir. Isocratic flow at 1 mL/min was used with a mobile phase consisting of a mixture of 10 mM ammonium acetate and 5% v/v acetonitrile at pH 6.5. Chromatographic separation was performed with a Phenomenex Luna (5μ) C18 column (4.6 × 150 mm) maintained at 25°C, after injection of 10 μL samples, with detection at 254 nm. Theophylline was used as an internal standard. A solution of 85 µg/mL theophylline was prepared in distilled deionized water generated by an EMD Millipore Milli-Q® water purification system. 50 µL of the acyclovir standard or sample solution (i.e., receptor solution or other extract) was added to 25 µl of the theophylline internal standard solution and vortexed to mix. A good linear relationship was observed between the area of the acyclovir peak and concentration for calibration standards from 0.048 to 300 µg/mL, with a high correlation coefficient (r^2^ = 0.9998). The method was precise (intra- and inter-day variation was < 5.0%) and accurate (mean recovery 99.5%). Content analysis was performed on both pump and tube products including the non-pump-dispensed product, that was scooped out from the back of a pump dispenser.

### *In vitro* Skin Permeation of Acyclovir from Zovirax® UK Acyclovir Cream, 5% (Pump and Tube)

#### Human Skin Preparation

Full thickness human skin samples obtained from patients (26–48 years old females) undergoing abdominoplasty at Brisbane (QLD) hospitals were refrigerated immediately after elective surgery. Sampling was approved by the Metro South and University of Queensland Human Research Ethics Committees (Approval number:2008001342) and was conducted in compliance with the guidelines of the National Health and Medical Research Council of Australia and of United States Food and Drug Administration’s Research In Human Subjects Committee. Epidermal sheets were obtained by first removing subcutaneous fat by dissection, then immersion of full thickness skin in water at 60°C for 1 min, after which the epidermis was teased off the dermis [22, 23]. The epidermis was air dried, then placed in a zip-lock bag and stored at -20°C until required.

### *In Vitro* Permeation Test (IVPT) Studies

*In vitro* permeation test (IVPT) studies across human epidermis were performed in glass unjacketed, static, vertical (Franz-type) diffusion cells (exposed skin area 1.33 cm^2^; receptor volume approximately 3.5 mL). Epidermal membranes (skin sections) were placed between the donor and receptor compartments and allowed to equilibrate for 1 h with the receptor solution (PBS pH 7.4 and 0.01% sodium azide) that was stirred continuously with a magnetic stirrer bar for 1 h. The unjacketed Franz-type diffusion cells were immersed in a water bath at 37 ± 0.5°C to maintain the skin surface temperature at approximately 32°C (verified by an infrared thermometer). PBS was placed in the donor compartment and the resistance across the epidermis was measured with a digital multimeter to assess skin barrier integrity. Skin sections exhibiting an electrical resistance of less than 20 kΩ.cm^2^ were rejected from the study [24]. The receptor was then refilled with approximately 3.5 mL of fresh pre-warmed receptor solution. The surface of the skin was blotted dry using Kim wipes and allowed to air dry shortly before dosing.

Pre-weighed amounts (approximately15 mg/cm^2^) of acyclovir cream (5%) from the pump and the tube were applied to the surface of the epidermis by spreading evenly with a 1 mL syringe plunger that was weighed before and after the spreading procedure. Two hundred μL samples of the receptor phase were withdrawn at various times over a 48 h period and replaced with equal volumes of fresh pre-warmed (37°C) PBS (pH 7.4) containing 0.01% sodium azide. Acyclovir concentrations in the receptor samples were analyzed by HPLC.

A total of 6 skin donors, with 3 skin replicates dosed per donor, were used for each acyclovir cream sample dispensed from the tube and the pump. The cumulative amount of acyclovir permeated through the epidermis (μg/cm^2^) versus time (h) was plotted and the flux (µg/cm^2^/h) through the epidermis was determined.

### Statistical Analysis

The cumulative amounts (µg/cm^2^) of acyclovir in the receptor chamber were determined over the entire period of testing and compared between the pump and tube.

Given the variable nature of human skin, the rate and extent of acyclovir bioavailability for the acyclovir cream, 5% product dispensed from the tube vs. the pump was compared based upon cutaneous pharmacokinetics endpoints using a mixed, reference-scaled criterion that resembles the bioequivalence assessment approach for highly variable drugs [9, 25]. According to this criterion, the within-reference standard deviation, ${S}_{WR}$, is used as a threshold value.

- If ${S}_{WR}\le 0.294$, a two-one sided 90% confidence interval is constructed for the difference of the means of the test and reference products, ${\mu }_{T}-{\mu }_{R}$ [26]. If this interval is contained within [0.80, 1.25], the two products are declared equivalent.
- If ${S}_{WR}>0.294$, a scaled confidence interval for ${({\mu }_{T}-{\mu }_{R})}^{2}-\theta {\sigma }_{WR}^{2}$ is constructed (where $\theta$ = $\frac{{(\text{ln}\left(1.25\right))}^{2}}{{(0.25)}^{2}}$), and its upper 95% bound is observed. Then the two products are declared equivalent if:
  1. The upper 95% bound of the scaled confidence interval is less than zero and
  2. The point-estimate (geometric mean ratio of test/reference) is contained within [0.80, 1.25].

## Results and Discussion

Zovirax® acyclovir cream, 5% is indicated for the treatment of recurrent herpes labialis (cold sores), and has been available in numerous countries for many years. It was initially introduced in a traditional tube but is now also available in a pump container closure system. Several studies have demonstrated that these pumps are often favored by patients, primarily for their ease of use [14, 15, 27].

We have previously demonstrated that the rheological characteristics and shear response affect the pharmaceutical equivalence, therapeutic equivalence, and sensorial equivalence of commercial topical products [28]. When the same topical semisolid product is dispensed from different packaging configurations, patients and physicians may expect that the product quality attributes and performance would be the same, or very similar, and the products dispensed from each packaging configuration may be assumed to be interchangeable. However, extrapolating from knowledge in the field of extrusion technology suggests that the packaging parameters and the process of dispensing may alter the formulation microstructure and thereby change its performance. Here, we demonstrated that the pump dispensing procedure did, indeed, alter Q3 attributes of the cream as well as the skin permeation profiles for acyclovir, compared to the same cream dispensed from a tube. The observed alterations in rheology caused by the pump dispensing process are particularly important in the case of semisolid products such as creams, that are susceptible to differential changes depending upon the manner of shearing, and specifically, the shear history associated with dispensing from the container closure system. In the pump product, the dispensing process involves the cream formulation transiting through a small orifice under pressure.

Our systematic examination of the quality attributes that could affect the microstructural properties of the formulation, including the number, size and shape of suspended drug particles, drug distribution and rheological characteristics has provided insights into how the dispensing process may have affected the product performance.

## Optical Microscopy Analysis

**Fig. 1:**
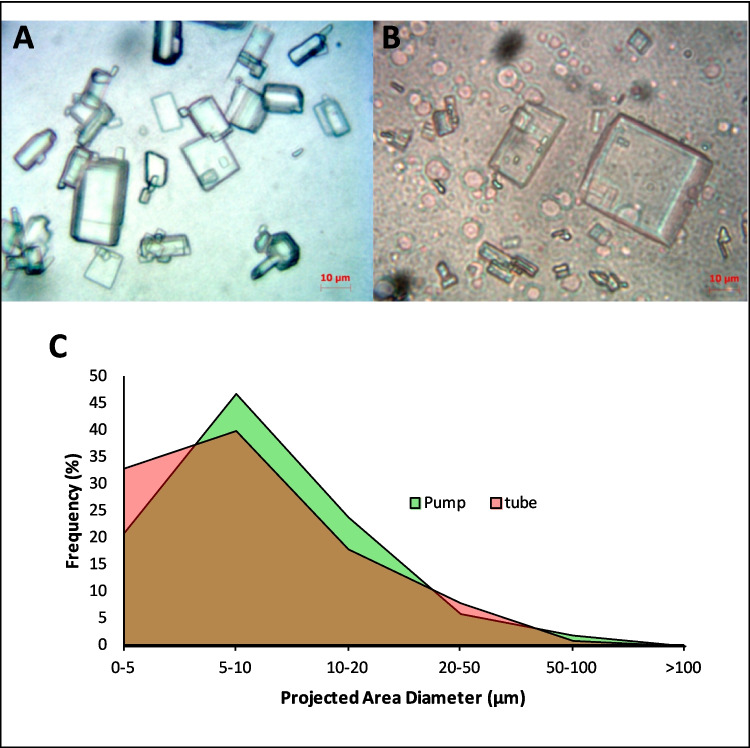
Optical microscopy image of (**A**) crystals in the cream dispensed from the tube, (**B**) crystals and oil globules in the cream dispensed from the pump with Sudan red staining and (**C**) projected area diameter of 100 particles in acyclovir cream dispensed from the pump and the tube.

Samples from both packaging configurations dispensed onto glass slides and subsequently visualized by optical light microscopy exhibited rectangular crystals are shown in Fig. 1A for the tube sample and in Fig. 1B for the pump. The slight reddish hue present in the image 1B is due to the addition of Sudan red which was added to differentiate globules from possible air bubbles. Image 1A was captured without the addition of Sudan red as no globules were evident in the sample. The particle size distributions were similar for the products dispensed from either the pump or the tube (Fig. 1C). The sample from the pump contained small globules consisting of a coalesced oil phase, identified by staining with Sudan red dye (Fig. 1B), whereas no coalesced oil phase globules were evident in the cream dispensed from the tube (Fig. 1A). As a control experiment, the body of the pump container closure system was cut open and the cream was removed without dispensing it through the pump nozzle (designated “no pump”). Here, no globules were detectable and the sample was similar to the sample from the tube, thus demonstrating that the globules were generated by the process of dispensing from the pump through the nozzle, presumably creating relatively higher shear on the cream compared to when dispensing from the pump.

### Identification of the Cream Components by Confocal Raman Microscopy

**Fig. 2:**
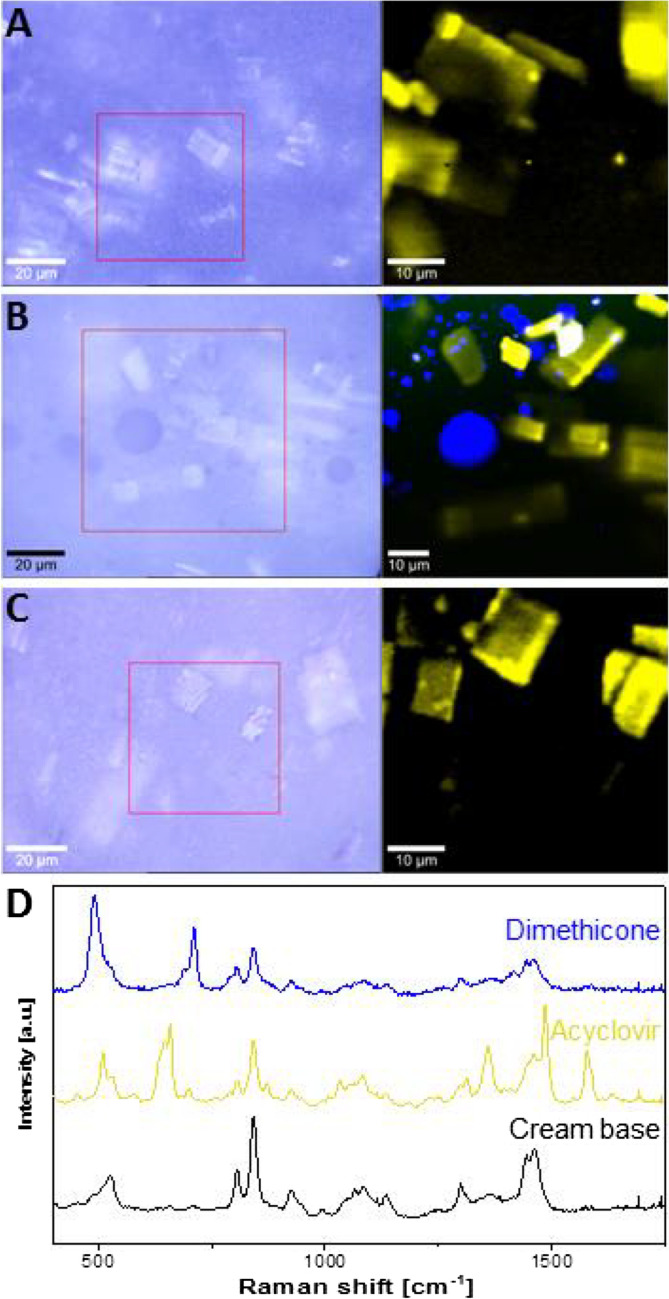
Confocal Raman microscopy analysis of acyclovir cream formulations from different packaging configurations and dispensing methods. Light microscopic images and corresponding Raman false color images of the areas indicated by the red square of samples dispensed from the tube (**A**), dispensed from the pump through the nozzle (**B**) and a sample removed from the inside of the pump container closure system without dispensing through the nozzle (**C**). The false color images show the cream base (black), acyclovir crystals (yellow) and dimethicone globules (blue): and the corresponding single spectra of these components (D).

Figure 2 shows light microscopic images and the corresponding false-colored Raman images of the same area. In the Raman images, yellow false-colored acyclovir crystals could be visualized in the three creams dispensed from the tube (Fig. 2A), dispensed from the pump through the nozzle (Fig. 2B) and removed by spatula from the pump that had been splayed open, without pump-dispensing (Fig. 2C), while the cream base is depicted in black. We have previously demonstrated the utility of confocal Raman microscopy (CRM) for the characterization of topical semisolid formulations [29]. Here CRM was used to identify the formulation ingredients present in the cream and to identify the globules from the pump that had been observed under optical microscopy. CRM involves irradiating the sample with a laser and detecting the frequency shift of scattered photons, to provide a “chemical fingerprint” for chemical identification. The technique allows for chemical characterization of pharmaceutical and biological substances without further sample preparation or the need for dyes and labels [30–32]. The acyclovir crystals show the same size and shape in the Raman and light microscopic images, and there are no obvious differences in the acyclovir crystals between the three samples. Notably, globules were only visible in the sample dispensed from the pump; these globules were readily visible in light microscopy images and were specifically identified as dimethicone globules by CRM (Fig. 2B, false-colored blue). No globules were detected in the sample from the tube (Fig. 2A) or in the sample removed by spatula from the pump container closure system (Fig. 2C), and Raman analysis indicated that no apparent disintegration (e.g., phase separation) of the cream base had occurred. The data suggest that the mechanical stress to the product, imposed by dispensing it via the nozzle of the pump, causes dimethicone to separate from the cream base and to form globules. Single spectra analysis of the cream base, drug crystals and dimethicone globules are depicted in Fig. 2D.

### Examination of the Internal Microstructure of the Creams by Electron Microscopy

**Fig. 3:**
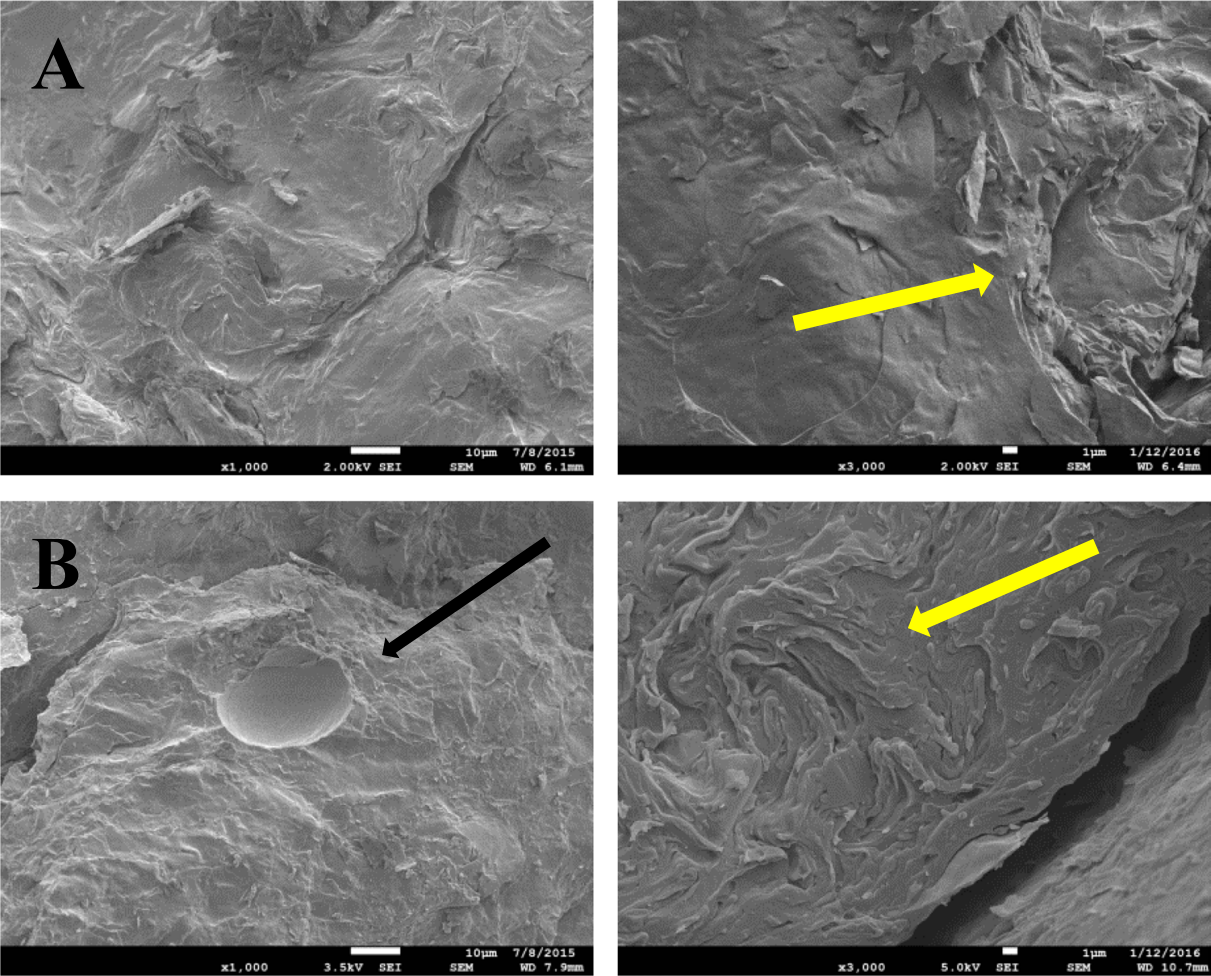
Cryo-SEM images showing the internal microstructure of the acyclovir cream dispensed from (**A**) tube (above images) and (**B**) pump (below images). Globules are shown by black arrow, and background lamellar fabric by yellow arrows). Scale bar 1 µm.

To corroborate the results obtained by optical microscopy and CRM, cryo-SEM micrographs were recorded after freeze fracture of the samples. Two images each are included for creams dispensed from a tube (Fig. 3A) and from a pump (Fig. 3B). As expected, dimethicone globules are observed in the cream sample dispensed from the pump (indicated by a black arrow), but not from the tube. The internal microstructure of the cream samples was lamellar apart from the presence of globules in the cream dispensed from the pump.

### Rheological Characterization

**Fig. 4:**
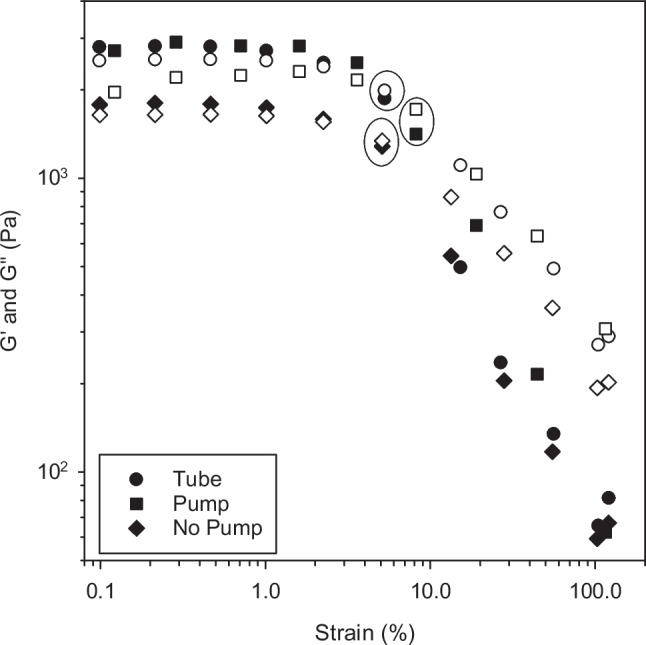
Storage (G’, filled symbols) and loss (G”, unfilled symbols) modulus against strain for the tube, pump and ‘no pump’. The strain at which G” crosses above G’ (circled) is taken as the yield point

**Fig. 5:**
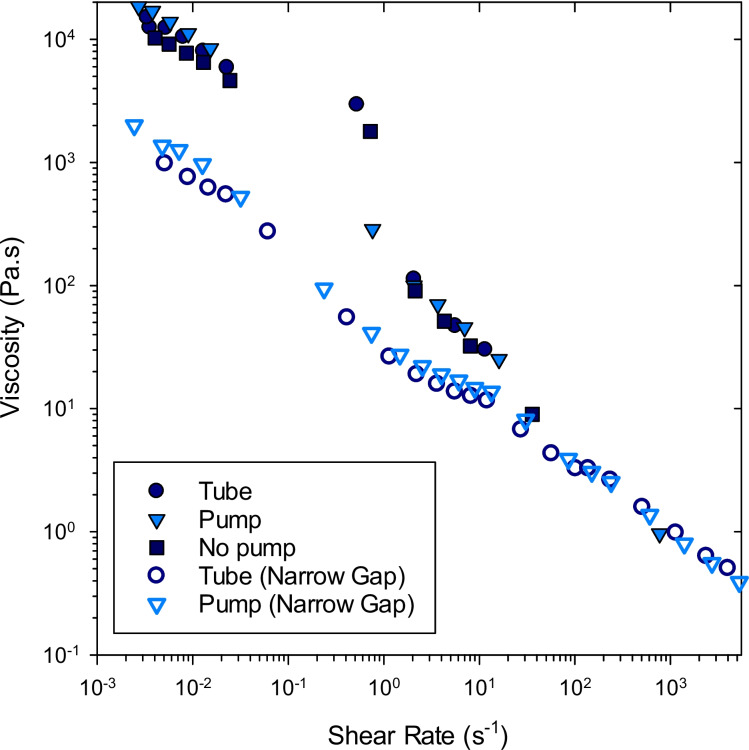
Viscosity vs. shear rate measured using wide gap (filled symbols) and narrow gap (open symbols) for acyclovir creams delivered from tube and pump, as well as removed from the pump without dispensing (No pump)

Our protocols to investigate the rheological behavior of the creams were designed to evaluate the effect of shear stress on the cream microstructure. Oscillatory rheology measured the yield stress relative to the stress experienced by the cream during dispensing. The shear stress that the cream experiences during dispensing through the nozzle of the pump causes the structural rearrangement seen in Fig. 1B. In addition, measuring the viscosity of the creams at wide (0.5 mm) and narrow (0.05 mm) gap evaluated the influence of the dispensing method on viscosity during initial application (wide gap) and spreading of the cream to a thin film (narrow gap). This is important as the viscosity of the cream affects the thickness of the product film on the skin and that may affect drug delivery [33–36]. To determine whether the rheological changes identified between the creams dispensed from pump and tube resulted from the dispensing procedure or were due to differences in the manufactured cream, measurements were also conducted on a cream sample that was removed from the pump container closure system without passing through the pump nozzle (labeled ‘No Pump’ in Figs. 4 and 5.

The storage modulus (G’) and loss modulus (G’’) were determined with increasing strain, as displayed in Fig. 4. In general, both acyclovir cream samples have a constant value of G’ up to approximately 1% strain. This is the linear viscoelastic region (LVR) in which any disturbance in the microstructure of the sample is reversible. For all acyclovir creams, G’ is greater than G’’ before the yield stress, indicating that the creams behave as viscoelastic solids. As the strain increases, G’’ exceeds G’, indicating that the acyclovir creams yield and begin to flow. The dynamic yield stress (the point where G’’ crosses above G’(35)) is circled in Fig. 4 for each of the creams at appropriate strain values [tube (5%); pump (8%); sample from within the tube removed with no pumping (5%)]. The corresponding yield stress values were 78 ± 1.3, 182 ± 0.6, 70 ± 10 Pa for the tube, pump and no pump samples, respectively. The yield stress is significantly different (*p* < 0.05) for the cream dispensed from the tube (78 ± 1.3 Pa) vs. that dispensed from the pump via the nozzle (182 ± 0.6 Pa) but not significantly different between the cream dispensed from the tube (78 ± 1.3 Pa) vs. the sample removed from within the pump, without pumping (70 ± 10 Pa), suggesting that the shear rate experienced by the cream when it is dispensed from the pump via the nozzle affects the yield stress.

The wall shear rate ($\dot{\gamma }$) is estimated using $\dot{\gamma}=\dot{8}v/d$, with velocity (*v*) and the diameter of the pipe (*d*). We estimate that during pump dispensing, the cream experiences an internal wall shear rates of ≈ 16 s^−1^, where the tube diameter is 1.5 mm. The cream is pumped through a tortuous path that includes an orifice blocked by a ball bearing over a spring and several small openings with an inner diameter of approximately 0.1 mm. This exposes the cream to shear rates of ≈ 250 s^−1^ that are significantly greater than the yield stress, resulting in the observed microstructural changes in the pump-dispensed cream. Exposure to shear rates above the yield stress of the fluid did not affect the viscosity of the cream at wide or narrow gap, as shown in Fig. 5, where there was no significant difference between the pump, tube and no pump samples at wide gap. However, at low shear rates there is an observed difference between the samples at wide and narrow gap, which results from the shear history experienced by the cream during loading on to the rheometer. The cream has effectively been pre-sheared at a higher rate when compressing the plates to narrow gaps. At narrow gap, there is no significant difference between the pump and tube samples suggesting that small changes in microstructure may not be identified by viscosity measurement. This observation has been made previously for low and high fat food products [37] and the use of tribology was successfully applied to differentiate between products of differing microstructure. In particular, it is a useful technique to identify the lubricating effects related to the product.

We conducted a tribological test (data not shown) to assess the role of large silicone oil globules on the lubrication properties of the pump-dispensed product. We found no differences in the friction coefficient between the creams dispensed from the tube and pump, suggesting that despite the potential lubricating properties of the silicone oil globules this was not the primary driver of the changes in the performance of the dispensed creams. We conclude that for the acyclovir creams studied, the use of oscillatory rheology to determine yield stress was most appropriate for differentiating the microstructure of the creams.

### *In vitro* Skin Permeation Testing Across Heat Separated Human Epidermis

**Fig. 6:**
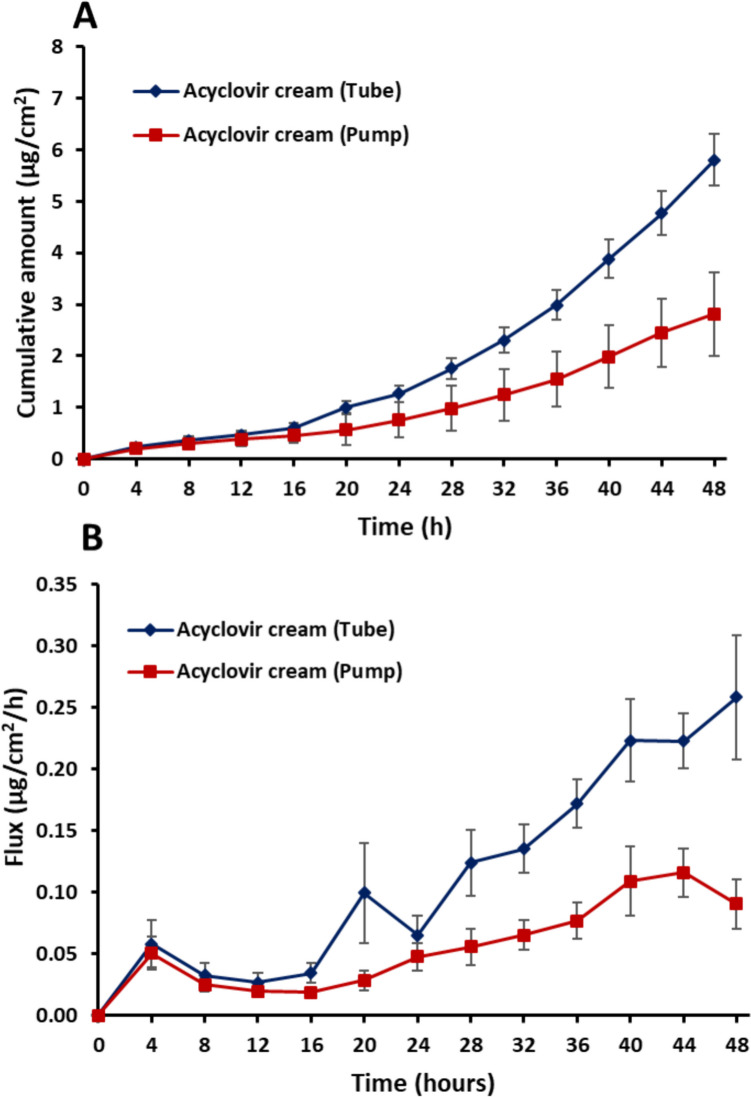
**(A)** Permeation profile and** (B)** flux curve of acyclovir from Zovirax® cream (UK)—tube and pump across heat separated human epidermis. Results are expressed as mean ± SEM, 6 skin donors, with 3 skin replicates dosed per donor per treatment.

*In vitr*o permeation tests (IVPT) using unjacketed static vertical Franz type diffusion cells were performed to assess and compare the bioavailability of acyclovir across heat separated human epidermis from a 15 mg/cm^2^ dose of the acyclovir cream, 5% dispensed from the tube and the pump packaging configurations of the Zovirax® UK product. The cumulative amount permeated (Fig. 6A) and flux rates (Fig. 6B) of acyclovir permeation through human epidermis was compared to evaluate the potential impact of the differences in the microstructure of the creams dispensed from the tube vs. the pump on acyclovir bioavailability. Permeation of acyclovir across human epidermis was significantly higher (*P* < 0.05) after it was dispensed from the tube compared to the pump (cumulative amount permeated over 48 h; 5.80 µg/cm^2^ ± 0.49 µg/cm^2^ versus 2.80 µg/cm^2^ ± 0.82 µg/cm^2^ for tube and pump, respectively). The average flux of acyclovir over 48 h from the tube (0.26 µg/cm^2^/h ± 0.05 µg/cm^2^/h) was also significantly higher (*P* < 0.0001) compared to the pump (0.09 µg/cm^2^/h ± 0.02 µg/cm^2^/h). A clear difference in rate and extent of delivery can be seen from 16 h onwards. These differences in product bioavailability may have clinical importance.

Analysis of the tube content, the pump-dispensed and non-pump-dispensed samples from the same container closure system showed less than 2% variability in acyclovir content, indicating that the lower permeability of acyclovir from the pump product compared to the tube product was not a result of lower acyclovir content in the pump. It is likely that the pump-induced coalescence of larger dimethicone globules identified by CRM influenced acyclovir permeation across human epidermis. Silicones such as dimethicone are included in topical semisolid products to impart water repellent properties to the formulation and skin surface, and enhance spreading and sensorial properties, and have been shown to influence skin permeation. For example, Montenegro *et al*. [38] observed that the silicone emulsifiers dimethicone and cyclomethicone permeated the skin to different levels and reduced the *in vitro* permeation of two sunscreens, octylmethoxycinnamate and butylmethoxydibenzoylmethane. We hypothesize that the permeation of acyclovir cream dispensed through the nozzle of a pump could be decreased due to the formation of a thin film of dimethicone on the skin surface that could impede drug partitioning into the stratum corneum.

The statistical analysis in Table I indicates that the rate and extent of acyclovir bioavailability for the acyclovir cream, 5% product dispensed from the tube vs. the pump was not shown to be bioequivalent based upon the results of the IVPT study conducted. While this quantitative analysis appears to be consistent with a qualitative assessment of the IVPT study results shown in Fig. 6, which appear to indicate an altered rate and extent of acyclovir bioavailability from the tube and the pump. However, the IVPT study may not have been adequately powered to demonstrate bioequivalence, if the two products actually were bioequivalent. Also, despite differences in acyclovir bioavailability, the products tested may still be therapeutically equivalent.

**Table I:** Statistical Analysis (SABE)

PK metric	Point Estimate	Between Donor SD	Within Ref SD	SABE UB	BE margin	90% ABE CI
Jmax	0.3605273	0.8889742	0.4793718	2.886082	1.25	(0.1735146, 0.7491007)
AUC	0.3906597	0.8199890	0.4908209	2.416449	1.25	(0.1989953, 0.7669279)
PK metric	**Point Estimate**	**Between Donor SD**	**Within Ref SD**	**SABE UB**	**BE margin**	**90% ABE CI**
Jmax	0.3605	0.8890	0.4794	2.8861	1.25	(0.1735, 0.7491)
AUC	0.3907	0.8199	0.4908	2.4164	1.25	(0.1990, 0.7669)

What is of greatest relevance to the aim of this study, is the identification of a potential failure mode for bioequivalence, and potentially for therapeutic equivalence. To our knowledge, this is the first study to report a difference in skin permeation from the same cream product dispensed from two different packaging configurations, and the results indicate that if the process of dispensing a formulation from the container closure system alters the microstructure of the cream, drug delivery into and through the skin may also be altered.

## Conclusions

In an ever-changing, consumer-driven market for pharmaceutical products, when customer preferences influence product development and marketing decisions to alter the container closure system (i.e., the packaging configuration), it may be prudent to consider the impact of the different container closure systems on the Q3 attributes of the dispensed product because it may have the potential to alter the performance of the product. One such example would be changing the container closure system of a product that was previously approved with a different container closure system. Topical semisolid products are influenced by a variety of factors, some leading to variation in product performance. We have shown that differential shear forces due to the mode of dispensing the product can affect both the microstructure of the product and the overall performance. Here, we have explored the important role played by Q3 attributes, notably product microstructure, in determining its performance. We showed that the force applied during product dispensing through the nozzle of a pump may be sufficient to alter the internal microstructure of the product, and that differences in the dispensing process may represent a failure mode for bioequivalence with the products. Hence, it may be prudent to characterize the impact of dispensing a product from different container closure systems on the Q3 attributes and performance of that product, to mitigate the risk of this potential failure mode for bioequivalence. This finding has important implications for the development of new products in alternative packaging configurations, as well as for the development of corresponding versions of generic products.

## Data Availability

All data generated or analysed during the study are included in this article.

## Abbreviations

BE: Bioequivalence
CRM: Confocal Raman microscopy
HPLC: High-performance liquid chromatography
IVPT: *In vitro* permeation test
PBS: Phosphate buffered saline
PDMS: Polydimethylsiloxane
QAs: Quality attributes
Q3: Physicochemical and Structural
QTPP: Quality Target Product Profile
SEM: Scanning electron microscopy
